# Postnatal Catch-Up Growth Programs Telomere Dynamics and Glucose Intolerance in Low Birth Weight Mice

**DOI:** 10.3390/ijms22073657

**Published:** 2021-04-01

**Authors:** Eva Pericuesta, Julia L. Gutiérrez-Arroyo, Maria J. Sánchez-Calabuig, Alfonso Gutiérrez-Adán

**Affiliations:** Departamento de Reproducción Animal, Instituto Nacional de Investigación y Tecnología Agraria y Alimentaria (INIA), 28040 Madrid, Spain; pcamacho@inia.es (E.P.); juliagut@ucm.es (J.L.G.-A.); mariasanchezcalabuig@gmail.com (M.J.S.-C.)

**Keywords:** fetal growth restriction, telomere shortening, sex, inbred, outbred

## Abstract

Low birth weight and rapid postnatal weight gain are independent predictors of obesity and diabetes in adult life, yet the molecular events involved in this process remain unknown. In inbred and outbred mice, this study examines natural intrauterine growth restriction (IUGR) in relation to body weight, telomere length (TL), glucose tolerance, and growth factor gene (*Igf1*, *Igf2*, *Insr*, *Igf1r*, and *Igf2r*) mRNA expression levels in the brain, liver, and muscle at 2- and 10 days of age and then at 3- and 9 months of age. At birth, ~15% of the animals showed IUGR, but by 3 and 9 months, half of these animals had regained the same weight as controls without IUGR (recuperated group). At 10 days, there was no difference in TL between animals undergoing IUGR and controls. However, by 3 and 9 months of age, the recuperated animals had shorter TL than the control and IUGR-non recuperated animals and also showed glucose intolerance. Further, compared to controls, *Igf1* and *Igf2* growth factor mRNA expression was lower in Day 2-IUGR mice, while *Igf2r* and *Insr* mRNA expression was higher in D10-IUGR animals. Moreover, at 3 months of age, only in the recuperated group were brain and liver *Igf1*, *Igf2*, *Insr*, and *Igf2r* expression levels higher than in the control and IUGR-non-recuperated groups. These data indicate that catch-up growth but not IUGR per se affects TL and glucose tolerance, and suggest a role in this latter process of insulin/insulin-like growth signaling pathway gene expression during early development.

## 1. Introduction

When an unborn baby (fetus) is smaller than expected for its gestational age, this is described as intrauterine growth restriction (IUGR), or fetal growth restriction, and is usually defined as an estimated weight less than the 10th percentile [1]. Fetal growth restriction is the outcome of genetic or environmental factors and is the end result of placental, fetal, maternal, and genetic factors, or any combination of these factors. In humans, IUGR resulting in low birth weight (<2.5 kg) followed by rapid postnatal catch-up growth increases the risk of the onset, in adulthood, of metabolic syndrome, type 2 diabetes, cardiovascular disease, and cancer. The idea was first described by Barker et al. [2] as “fetal origins of adult disease,” and later supported by a hypothesis known as Developmental Origins of Adult Health and Disease (DOHaD). According to this hypothesis, suboptimal conditions during critical periods of development are key factors affecting developmental programming, and these factors can not only affect adult health and disease, but also the rate of aging per se [3]. In effect, ageing is a major risk factor for the majority of these diseases associated with DOHaD. Among the factors identified to increase the risk of adult metabolic disturbances is postnatal catch-up growth in low birthweight individuals [4]. However, the mechanisms that lead to this risk have not been clarified. Some studies have shown that while a lower birth weight is important, the rate at which an individual regains weight during infancy is a critical factor affecting the risk of later developing obesity and metabolic syndrome [5,6]. This is important as the classic notion upheld by pediatricians is that IUGR newborns should grow quickly to reach the size of normal birthweight babies, yet there is new evidence to indicate that rapid catch-up growth in IUGR babies predisposes them to a greater risk of subsequent obesity and other metabolic diseases.

There is also evidence of the great impact telomeres have on health, the dynamics of aging, longevity, and the development of many genetic diseases [7]. Recently, fetal telomere length (TL) has been linked to prenatal exposure to tobacco smoke, maternal stress, maternal disease, and nutritional and sleeping disorders during pregnancy [8]. In humans, leukocyte TL was found longer in small-for-gestational age neonates than in controls [9,10]. This is surprising, as usually small-for-gestational age babies will have an increased risk of developing metabolic and cardiovascular diseases in adulthood and these diseases are associated with short telomeres [10,11]. Interestingly, in the study by Laganovic et al. [9] it was observed in young men that all premature birth groups underwent faster telomere shortening compared to controls. Moreover, shorter TL was observed in placenta samples collected from women with pregnancies complicated by IUGR [12]. In a recent meta-analysis, preterm birth was associated with longer birth TL, but IUGR was not related to birth telomere length [13]. In animal models in which IUGR is induced by a diet low in protein during gestation, raising postnatal nutrition to normal levels in a protein-restricted fetus causes rapid growth associated with subsequent obesity [14] and this seems to affect TL in rat offspring [15,16]. However, this low diet protein IUGR model is not representative of the situation in developed countries, as mothers are typically not severely protein-deprived. For this reason, in the present study we explore another animal model (natural IUGR) that does not involve severely limiting maternal nutrient intake through protein or calorie restriction.

Insulin-like growth factor-1 (IGF-1) signaling influences mammalian aging and related disease [17]. During pregnancy, IGF-1 signaling regulates fetal and placental development and controls cell differentiation into adult tissues [17]. Maternal IGF-1 levels decline in the first trimester of pregnancy, increase by more than 40% over weeks 17–24 [18], and thereafter decrease again to pre-partum levels rapidly after delivery [19] indicating the regulation of IGF-1 levels in relation to the body’s needs. In rodents and humans, *IGF2* transcripts are found in all fetal tissues, and their expression declines early in the postnatal period [20]. The control of insulin-like growth factor-1 receptor (*Igf1r)* expression occurs mainly at the level of transcription [21]. IGF1R and insulin receptor (INSR) can form IGF1R/INSR hybrid receptors (HybR) consisting of one molecule of IGF1R and one molecule of INSR. HybR can be activated by both IGF1 and insulin, and like holo-IGF1R promotes cell proliferation and glucose uptake, but the specific signaling and functions of HybR are largely unknown [22].

In this study, we examined whether natural IUGR leading to the delivery of very low birthweight mouse pups could trigger fetal programming of the metabolic syndrome cascade of increased weight and glucose intolerance in adults. Inbred and outbred mice were used to determine if our IUGR model has genetic and/or non-genetic components. We also analyzed the relationship between this model of IUGR and possible molecular mechanisms such as initial telomere length, telomere attrition rate during early development, and the expression of insulin receptor and insulin-like growth factors and receptors. 

## 2. Results

### 2.1. IUGR Mice Generation and Weight at Three Months of Age

To identify the animals that had suffered IUGR, the weight of the animals was determined on D2 of birth. To this end, we used two strains of mice, a consanguineous strain (C57BL/6NHsd denominated as B6) and a non-consanguineous strain (CD1). The latter has a genetic profile that more resembles that of other mammals including humans. IUGR was defined following the same criterion used in humans, namely a weight less than 10 percent of the expected weight for age [1]. In most of the litters, 1 or 2 pups were below the 10% percentile of weight (Supplementary Figures S1 and S2). Based on D2 weight of the first 150 B6 or CD1 mice born, weights below the 10th percentile, were on average 1.52 g or 1.65 g for males respectively, and 1.47 g or 1.58 g for females respectively. 

**Figure 1:**
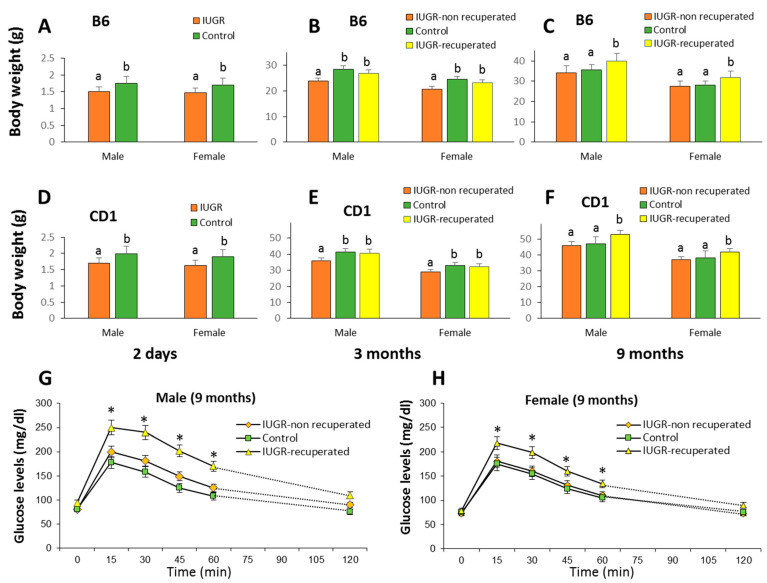
Weights and glucose tolerance of the mice used in this study. Weight at 2 days of age is indicated in B6 (**A**) and CD1 (**D**) mice with IUGR and controls of both sexes. Also indicated are weights at 3 months of age of the B6 (**B**) and CD1 (**E**) mice, and at 9 months of age of the B6 (**C**) and CD1 (**F**) mice. Weights are provided for all animals with IUGR, without IUGR (controls) and those with IUGR that at three months showed a similar weight to controls (IUGR-recuperated). Glucose tolerance test (1.5 g glucose/kg body weight, intraperitoneal) in unrestrained 9-month-old male (**G**) and female (**H**) mice after an overnight fast (n = 7 per group). Blood glucose measurements were taken every 15 min for 120 min with a blood glucose monitor (Accu-Chek). Symptoms of metabolic syndrome (glucose intolerance) were observed in IUGR-recuperated mice but not in the IUGR non-recuperated mice. Results provided as the mean ± SD. a,b different letters indicate significant differences (*p* < 0.05). *, *p* < 0.05 versus control mice.

CD1 mice weighed more than B6 mice, and in both strains, the mean weight of males was slightly higher than that of females, although not significantly (Figure 1A,D). In litters in which one animal with IUGR was identified, a medium-weight animal (control) was also selected and in both of them we marked their tails with one or two dots of ink, respectively; at 10 days, tattoos were also made on the fingers to individually identify each mouse with or without IUGR. In total, we used twenty-seven litters of B6 mice to obtained 18 males and 17 females that were marked as IUGR, and thirty-two litters of CD1 mice to obtained 17 males and 15 females as IUGR. Each litter was kept with its mother in an individual cage and at 10 days of age animals were tattooed and tail biopsies were taken of the IUGR animals and controls and stored at −20 °C until analysis. 

Animals were weaned at 21 days and weighed at 3 months of age which corresponds approximately to 17 years in humans [23]. In both strains and both sexes, about half of the animals with IUGR maintained a weight lower than the 20th percentile of the control population (9 B6 males and 8 B6 females, and 8 CD1 males and 7 CD1 females). The remaining animals with IUGR (approximately half) attained a weight greater than the 20th percentile similar to the controls (Figure 1B,E). At 3 months of age, a tail biopsy of the three groups of mice (non-IUGR, IUGR-recuperated, IUGR-non recuperated) was performed again and tissue samples were frozen at −20 °C. At 9 months of age, a similar pattern was observed, but in this case IUGR-recuperated group has a weight greater than control and IUGR-non recuperated groups (Figure 1C,F). 

Random-feeding blood glucose levels did not differ among groups (data not shown) but fasting glucose was higher in the IUGR-recuperated group both of male and female B6 mice suggesting a prediabetic state of mice that had regained weight (Figure 1G,H).

### 2.2. Dynamics of TL in IUGR and Control Mice between Two Days and Three Months of Age

**Figure 2:**
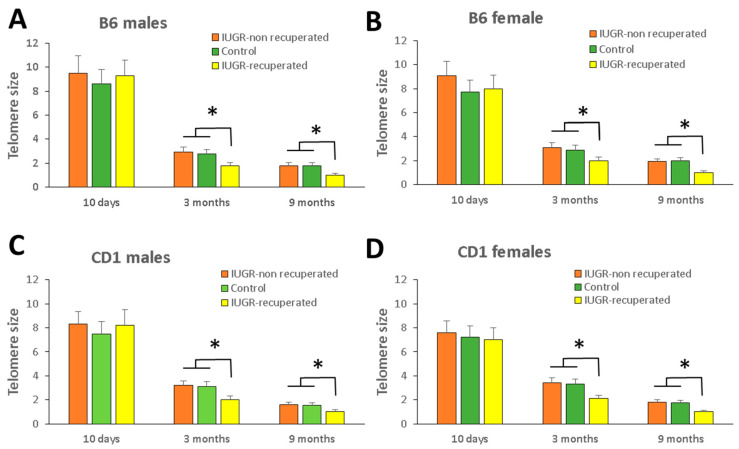
Relative telomere length. Telomere size determined at 10 days, 3- and 9-months of age in mice of strains B6 (**A**,**B**) and CD1 (**C**,**D**) with IUGR and controls of both sexes. Note that at 3- and 9 months of age, both in male and female mice, the IUGR animals reaching the weight of control animals (IUGR-recuperated) have a shorter telomere length than the other groups. Results provided as the mean ± SD. *, *p* < 0.05 versus control and IUGR-non recuperated mice.

Related to telomere length, at 10 days of age, there were no significant differences between IUGR and control animals or between males and females. In both strains the differences were not significant (Figure 2). 

When we examined TL at 10 days and 3 and 9 months, in both male and female, we observed a large decrease in length between D10 and 3 months (Figure 2) but no differences were noted from 3 to 9 months of age. At 3 and 9 months, animals born with IUGR that still maintained a weight lower than their corresponding controls showed a telomere length similar to controls. In contrast, the animals born with IUGR that had regained weight showed no differences in weight to controls but had a shorter telomere length in both strains than the other two groups (Figure 2).

### 2.3. Expression of Insulin-Like Growth Factors and Receptors in the Brains, Livers and Muscles of IUGR Mice

The expression was examined of several genes related to insulin-signaling pathway activation (insulin receptor, *Insr*), mediating growth and development (insulin-like growth factor 1, *Igf1*; insulin-like growth factor 1 receptor, *Igf1r*; insulin-like growth factor 2, *Igf2*; insulin-like growth factor 2 receptor, *Igf2r*), and telomerase activity (telomerase reverse transcriptase, *Tert*). For the analysis of gene expression of mice of 2 and 10 days, new litters of B6 mice were produced, and 3 females for control and IUGR group, obtained from different litters, were used.

**Figure 3:**
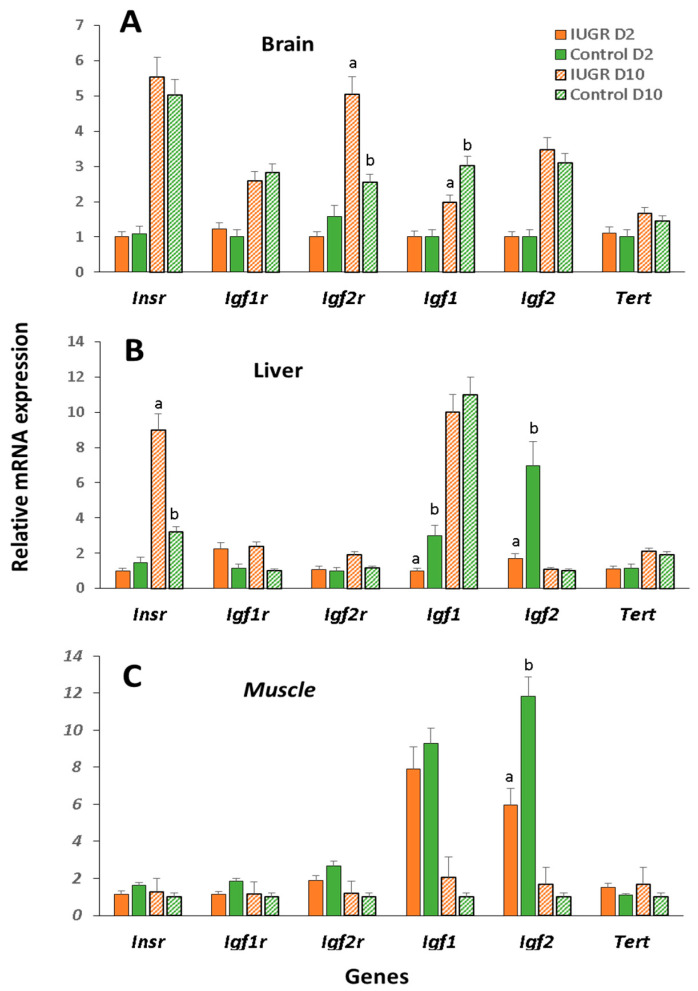
Relative mRNA expression of insulin receptor and insulin-like growth factors and receptors in IUGR B6 female mice. Relative expression levels recorded on Day 2 and Day 10 in the IUGR and control group in brain (**A**), liver (**B**), and muscle (**C**). Data expressed relative to levels recorded for the housekeeping genes *Actb* and *H2afz*. Results are provided as the mean ± SD. a,b different letters indicate significant differences between IUGR and control groups on Day 2 or Day 10 of life (*p* < 0.05).

In 2-day-old animals, we found greater expression levels of *Igf1*, *Igf2* in the liver, and *Igf2* in the muscle of IUGR group mice than the control mice (Figure 3B,C). At 10 days of age, differences were only observed in the brain, *Igfr2* expression being higher in the IUGR than control group, whereas *Igf1* expression was higher in the control than IUGR group (Figure 3A). In addition, liver *Insr* expression was higher in the IUGR group (Figure 3B).

**Figure 4:**
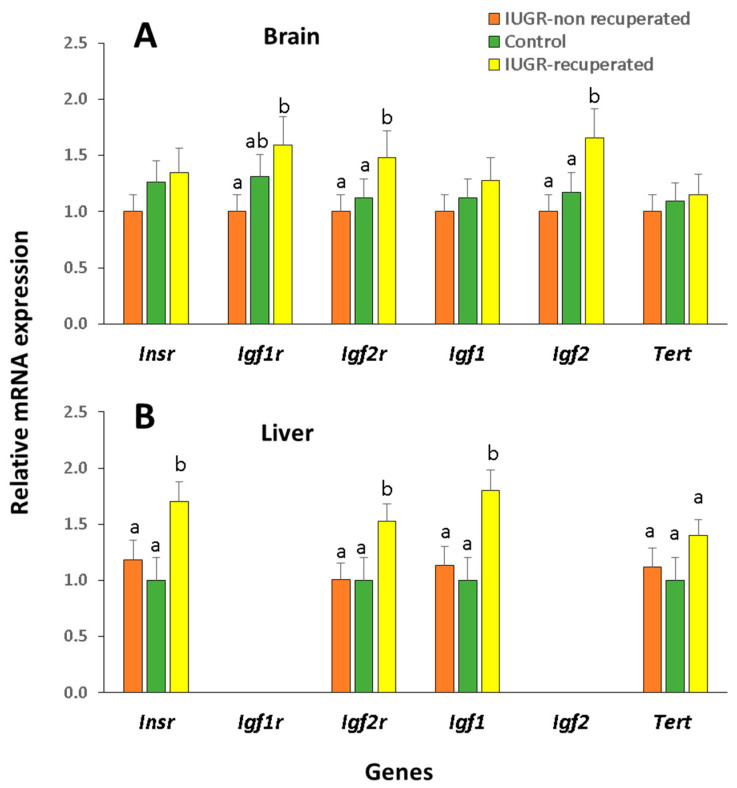
Relative mRNA expression of insulin receptor and insulin-like growth factors and receptors in IUGR B6 female animals. Relative expression levels recorded at 3 months of age in the IUGR-non recuperated, control, and IUGR-recuperated groups in brain (**A**) and liver (**B**). Data expressed relative to levels recorded in the housekeeping genes *Actb* and *H2afz*. Results are provided as the mean ± SD. a,b different letters indicate significant differences between groups (*p* < 0.05).

For the analysis of gene expression at 3 months of age, we used 3 females of strain B6 from the control, IUGR-non recuperated, and IUGR-recuperated groups (which we previously obtained in the experiment where we measured weight and telomeres). Because the muscle expression of all the genes analyzed was very low, so we only examined their brain and liver expression (Figure 4). Of the three experimental groups at 3 months only IUGR-recuperated showed the increased expression of *Igf1r*, *Igf2r*, and *Igf2* genes in the brain, and of *Igf2r*, *Insr*, *Igf1*, and *Tert* genes in the liver. No differences in *Tert* mRNA expression between groups were found in the brain, but in the liver there was a negative correlation between the size of telomeres and *Tert* mRNA expression between IUGR-recuperated and Control or IUGR-non recuperated groups. 

## 3. Discussion

In this study, we found that the IUGR mice that regained weight after three months showed shorter telomeres than control non-IUGR mice and IUGR-non recuperated mice. Moreover, only these recuperated IUGR mice showed the modified expression of genes related to the insulin/insulin-like growth factor signaling pathway at 3 months of age, along with glucose intolerance at 9 months of age. Our results suggest that the modified expression of *Ifg1* and *Igf2* at two days, and of *Igf1*, *Insr*, and *Igf2r* at ten days, could be associated with later telomere shortening and alterations in both glucose tolerance and the expression of insulin/insulin-like growth factor signaling pathway genes.

IUGR is a syndrome of multifactorial origin with different consequences. This means that different animal models are needed to improve biological screening for the consequences of fetal growth restriction and to determine molecular mechanisms and therapies. The main advantages of mouse models for this purpose are their short period of gestation, small size, and easy maintenance. Several models of fetal growth restriction affecting mothers have been described: transgenic mice, caloric restriction, protein restriction (9% vs. 20%), crowded uterine horn through unilateral ovariectomy pre-pregnancy, and uterine artery ligation or occlusion [24]. Here we identified cases of natural IUGR not affecting the mother. We believe this new model better reproduces fetal growth restriction in human pregnancy caused by placental insufficiency. This model is free of the effects of a deficient nutrition or health status of the mother, and lacks surgical interventions to remove an ovary or uterine artery ligation during pregnancy. As shown in Supplementary Figure S1, the cause of natural IUGR in our models using both strains of mice is the implantation position of the fetus, as placenta development and placental transport may be reduced when there is little space between fetuses, and this will limit concentrations of nutrients and oxygen reaching the fetus. We propose this model could resemble IUGR in humans caused by reduced placental blood flow and thus the active transport of specific nutrients [25].

In relation to the strain of mice used, we observed similar variation in birth weight and similar subsequent behavior of weight and telomeres in both the consanguineous (B6) and non-consanguineous (CD1) strains. This determines that causes of IUGR and subsequent postnatal effects cannot be due to genetic variability and thus must be the consequence of epigenetics and environmental variability (e.g., position and proximity of embryo implantation in the uterus).

We found that not all mice with IUGR undergo TL shortening at 3 months, only those showing rapid weight gain. These results coincide with Baker’s hypothesis [2], as only animals able to adapt as fetuses to the suboptimal conditions produced by IUGR during the postnatal stage, when food is available *at libitum*, undergo rapid catch-up growth and telomere shortening. While it is unknown why only some IUGR animals do this, other authors have also observed that only a fraction of a given population will show developmental alterations and rapid weight regain [26]. Telomere shortening is considered a characteristic of aging [27]. In effect, short telomeres are sufficient to cause the body to age, induce common chronic diseases and decrease lifespan [27]. Telomere biology could play a role as a signaling mechanism mediating the effects of IUGR, with subsequent health and disease consequences. It could be that stress suffered in utero causes TL shortening after birth, but we observed similar TLs at 10 days of age in both low- and normal weight IUGR animals. Another possible mechanism could be that fetal stress incurred during suboptimal development conditions modifies or programs telomere biology at a later stage of development and, as a consequence, produces a reduction in TL, as observed in our experiments. However, IUGR animals that do not regain weight were noted here to not undergo TL shortening, so we propose as a more likely mechanism that in animals that suffer IUGR at birth and then gain weight quickly, this rapid catch-up growth may be the consequence of a higher rate of cell replication determining that cells reduce TL. Alternatively, this reduction could be a consequence of the inflammatory process or endocrine stress that produces rapid weight gain. 

Results in humans indicate that severe stress during fetal development reduces telomere size at 25 years of age [28]. It has been also observed in humans that the telomere size of smaller-than-average babies is not affected at birth [29], as observed in this work, or at 11 years of age [30]. In addition, disturbances in fetal development have been linked to postnatal telomere length dynamics [31]. In zebrafish and wild birds, telomere size at 25 days of age was found to predict life expectancy [32,33]. This supports the relationship between telomere length and lifespan, and highlights a need to identify the factors that determine telomere length in early life.

Some authors propose that IUGR-induced changes include long-term epigenetic modifications to key genes that produce life-long metabolic and endocrine abnormalities such as altered concentrations of GH, insulin, and IGFs [34]. At 2 days of age, our IUGR animals showed lower expression in the liver of *Igf1* and *Igf2*, and in the muscle of *Igf2*. In contrast, at 10 days, IUGR animals featured higher liver expression of *Insr* and higher brain expression of *Igf2r*. We hypothesize that the high expression of receptors in the IUGR group may be a mechanism destined to offset the lower production of IGFs detected on Day 2 of life. The situation worsens when on Day 10 there is no longer a difference in IGF expression such that the excess of receptors will induce an abnormally greater response.

Interestingly, at three months of age, only the group of mice that were born underweight and later regained a normal weight featured alterations in the brain expression of *Igf2*, *Igf1r*, and *Igf2r*, and liver expression of *Igf1*, *Igf2r*, and *Insr*. *Igf2* is normally expressed in the fetal state, so its expression in postnatal stages of development may indicate its abnormal regulation. In addition, its *Igf2r* receptor is also overexpressed, so it could be that its expression is altered, perhaps through a mechanism designed to compensate for the lower birth weight. The insulin/insulin-like growth factor signaling pathway is a major conserved regulator of aging. It has been reported that IGF1R levels in the brain, but not in peripheral tissues, negatively correlate with longevity in 16 rodent species [35]. The higher expression of *Igf1* in the liver of our IUGR-recuperated animals warrants consideration. It has been reported that IGF1 secretion by the liver is dependent on growth hormone signaling and high levels show an inverse correlation with lifespan [36]. IGF1 has been also described to induce telomere shortening in human skin fibroblasts [37]. 

Our results suggest that physiological stress during the intrauterine period of development responsible for IUGR modifies insulin/insulin-like growth factor signaling, and these signals may have a relationship with telomere shortening. In turn, this induces postnatal catch-up growth and programming of the telomere biology system, increasing disease susceptibility over the organism’s life span.

## 4. Materials and Methods

### 4.1. Animals

All reagents were purchased from Sigma (Madrid, Spain) unless indicated otherwise. Animal experiments were conducted according to European legislation. All study protocols were approved by the Ethics Committee on Animal Experimentation of the INIA (Madrid, Spain) (21 September 2015) and were registered at the Direccion General de Agricultura y Ganaderia de la Comunidad de Madrid (Spain) (PROEX 261/15, 4 November 2015). For experiments we used CD-1 and B6 (C57BL/6NHsd) mice (Envigo, Envigo RMS Spain S.L.). All mice were kept in an animal facility with a controlled temperature and photoperiod (23 ± 1 °C and a cycle of 14 h light:10 h darkness) and had access to water and food ad libitum. Adult mice (3–4 months of age, virgin female mice) were bred for 2 weeks and then transferred to individual cages. Eight males of both strains were used in all crosses. Dams delivered naturally, fostered their own pups, and only litters with 5 to 7 (for inbred B6, average litter size of 6.4 in the mouse facility) and 7 to 9 (for outbreed CD1, average litter size of 8.6 in the mouse facility) animals were used in the experiments. 

Neonatal growth restriction or microsomia was defined as a gender-specific weight on postnatal Day 2 (D2) above the 10th percentile for the colony [38]. To lessen the risk of rejection, nests were undisturbed for 24 h after delivery, and initial pup weights were obtained on postnatal D2. Pups whose weight on D2 was under the 10th percentile (IUGR) or within the 50th percentile (control) were marked with indelible ink, and they were later tattooed to identify each animal on Day 10. For every microsomic mouse detected (18 B6 males and 17 B6 females and 17 CD1 males and 15 CD1 females), a corresponding littermate control mouse was identified (14 and 15 B6 males and females respectively, and 15 and 14 CD1 males and females, respectively). No more than two microsomic and control mice were obtained from a given litter. Pups were weaned to postnatal D21 and kept in groups of 5 animals for 3 months, when their weights were recorded. Day 2 and Day 10 mice were euthanized by decapitation with surgical scissors, and adult mice were euthanized with CO_2_.

### 4.2. DNA Sources

For the telomere size assay, DNA was extracted from mouse tail at 10 days and 3 months of age according to the standard procedures. Briefly, 30 µL of a dilution of proteinase K (1.25 mg/uL) in Tris NaCl ethylenediaminetetraacetic acid (EDTA) buffer (STE) was kept overnight at 55 °C, then cooled to 4 °C and diluted in a final volume of 300 µL of DNase-free water. After digestion, genomic DNA was extracted using a phenol-chloroform protocol followed by isopropanol precipitation and the pellet dissolved in 200 µL of DNase-free water and then quantified by spectrophotometry using a BioPhotometer (Eppendorf, Madrid, Spain) and diluted to a final concentration of 50 ng/μL before determining telomere length by qPCR. DNA samples were stored in the freezer at −20 °C.

### 4.3. Telomere Length 

Relative TL was determined using a described real-time quantitative PCR method [39] with minor modifications, in which telomeric DNA is amplified using specially designed primers [40]. Quantities of telomere repeats were normalized to the DNA quantity present in the sample (determined as multicopy gene 18S rRNA quantity) by the comparative Ct method [41]. Two microliters of the extracted DNA were used for each 18 uL of mix containing GoTaq^®^ Green Master Mix 2×, as described in the manufacturer’s instructions for the qPCR reaction conducted in a Rotorgene 6000 Real Time Cycler (Corbett Research, Sydney, Australia) (94 °C 3 min followed by 40 cycles of 94 °C 10 s, 60 °C 30 s, and 72 °C 30 s). For primer sequences see Supplementary Table S1.

### 4.4. Blood Glucose 

B6 mice were challenged with a glucose tolerance test at 9 months of age [42]. For this test, animals were transferred to a clean cage and fasted overnight (14 h) with ad libitum access to water and subjected to the test in the morning. Whole-blood b-D-glucose levels were determined using a standard handheld glucometer (Glucocard G-sensor, Arkray Factory, Inc., Shiga, Japan) in blood samples (2 µL/measurement) collected from the tail tip. Following baseline glucose measurements, mice were intraperitoneal injected with glucose (20% solution, 1.5 mg/g) and blood glucose readings were then taken 15, 60, and 120 min post-injection. Food was reintroduced immediately after the last reading. The area under the curve (AUC) corresponding to plasma glucose levels (AUCglucose) was calculated for the entire 120-min study period using the trapezoid rule.

### 4.5. Messenger RNA Levels (RT-qPCR) 

Total RNA was extracted from brain, liver, and muscle of B6 female animals using the kit TRIsure (Bioline, Reus, Spain) and the RNA eluted with RNA-free water. RNA integrity of total RNA was done by running an aliquot of the RNA sample on a denaturing agarose gel. RNA samples were stored in the freezer at −80 °C. After extraction, the RT reaction was carried out using poly (T) primers, random primers, and MMLV High Performance Reverse Transcriptase enzyme (Epicenter Technologies Corp., Madison, WI, USA) to prime the RT reaction and generate cDNA. Tubes were heated to 70 °C for 5 min to denature the secondary RNA structure and the RT reaction was completed with the addition of 100 units of reverse transcriptase. The mixture was incubated at 42 °C for 60 min to allow the RT of RNA, which was followed by incubation at 70 °C for 10 min to denature the RT enzyme. cDNA from three animals were set up for each experimental group with two technical replicates for all genes of interest. PCR was performed by adding a 2 μL aliquot of each sample (50 ng of cDNA) to the PCR mix containing specific primers to amplify the genes of interest. Primer sequences are provided in Supplementary Table S1. Expression levels were normalized against that of the endogenous controls *H2afz* and *Actb* as described previously [43,44]. PCR conditions were optimized to achieve efficiencies close to 1. The comparative cycle threshold (CT) method was used to quantify expression levels [41]. 

### 4.6. Statistical Analysis

Data were analyzed using the SigmaStat 4.0 (Jandel Scientific, San Rafael, CA, USA) software package. One-way ANOVA (followed by multiple pair-wise comparisons using Tukey’s test) was used for the analysis of mRNA expression, animal weight, and TL differences between groups. Before ANOVA, normal distribution with equal variances per group was analyzed, and when the data were not normally distributed a logarithmic transformation of data was done. Significance was set at *p* < 0.05.

## Supplementary Materials

The following are available online at https://www.mdpi.com/article/10.3390/ijms22073657/s1, Figure S1: Images of a 19-day-old litter of B6 mice, Figure S2: Images of two Day 2 litter of B6 mice, Table S1: Primers used in the gene expression and telomere size analyses.
