# Supplementary material for: Postnatal Catch-Up Growth Programs Telomere Dynamics and Glucose Intolerance in Low Birth Weight Mice
Source: Int J Mol Sci. 2021 Apr 1;22(7):3657. doi: 10.3390/ijms22073657 (PMC8037520; doi:10.3390/ijms22073657)
Supplement: Supplementary file 1 [file ijms-22-03657-s001.zip › Supplementary_table 1 primers.docx]

**Supplementary Table S1. Primers used in the gene expression and telomere size analyses**

| **Target** | **Forward primer (5' to 3')** | **Reverse primer (5' to 3')** |
| --- | --- | --- |
| **RT-qPCR gene expression analysis** | |  |
| *lgf1* | TCATGTCGTCTTCACACCTCTTCT | CCACACACGAACTGAAGAGCAT |
| *lgf2* | ACAACTTCGATTTGAACCACATTC | GAGAGCTCAAACCATGCAAACT |
| *lgf1r* | GTGGGGGCTCGTGTTTCTC | GATCACCGTGCAGTTTTCCA |
| *lgf2r* | GGGAAGCTGTTGACTCCAAAA | GCAGCCCATAGTGGTGTTGAA |
| *Insr* | ATGGGCTTCGGGAGAGGAT | GGATGTCCATACCAGGGCAC |
| *H2afz* | AGGACGACTAGCCATGGACGTGTG | CCACCACCAGCAATTGTAGCCTTG |
| *Actb* | CATTGCTGACAGGATGCAGAAGG | TGCTGGAAGGTGGACAGTGAGG |
|  |  |  |
| **qPCR quantification of telomere size** | |  |
| *Tel 1b* | CGGTTTGTTTGGGTTTGGGTTTGGGTTTGGGTTTGGGTT | |
| *Tel 2b* | GGCTTGCCTTACCCTTACCCTTACCCTTACCCTTACCCT | |
| *Rn18s* | AGAAACGGCTACCACATCCAA | CCTGTATTGTTATTTTTCGTCACTACCT |
