# Supplementary material for: Postnatal Catch-Up Growth Programs Telomere Dynamics and Glucose Intolerance in Low Birth Weight Mice
Source: Int J Mol Sci. 2021 Apr 1;22(7):3657. doi: 10.3390/ijms22073657 (PMC8037520; doi:10.3390/ijms22073657)
Supplement: Supplementary file 1 [file ijms-22-03657-s001.zip › Supp Fig 1.pptx]

## Slide 1
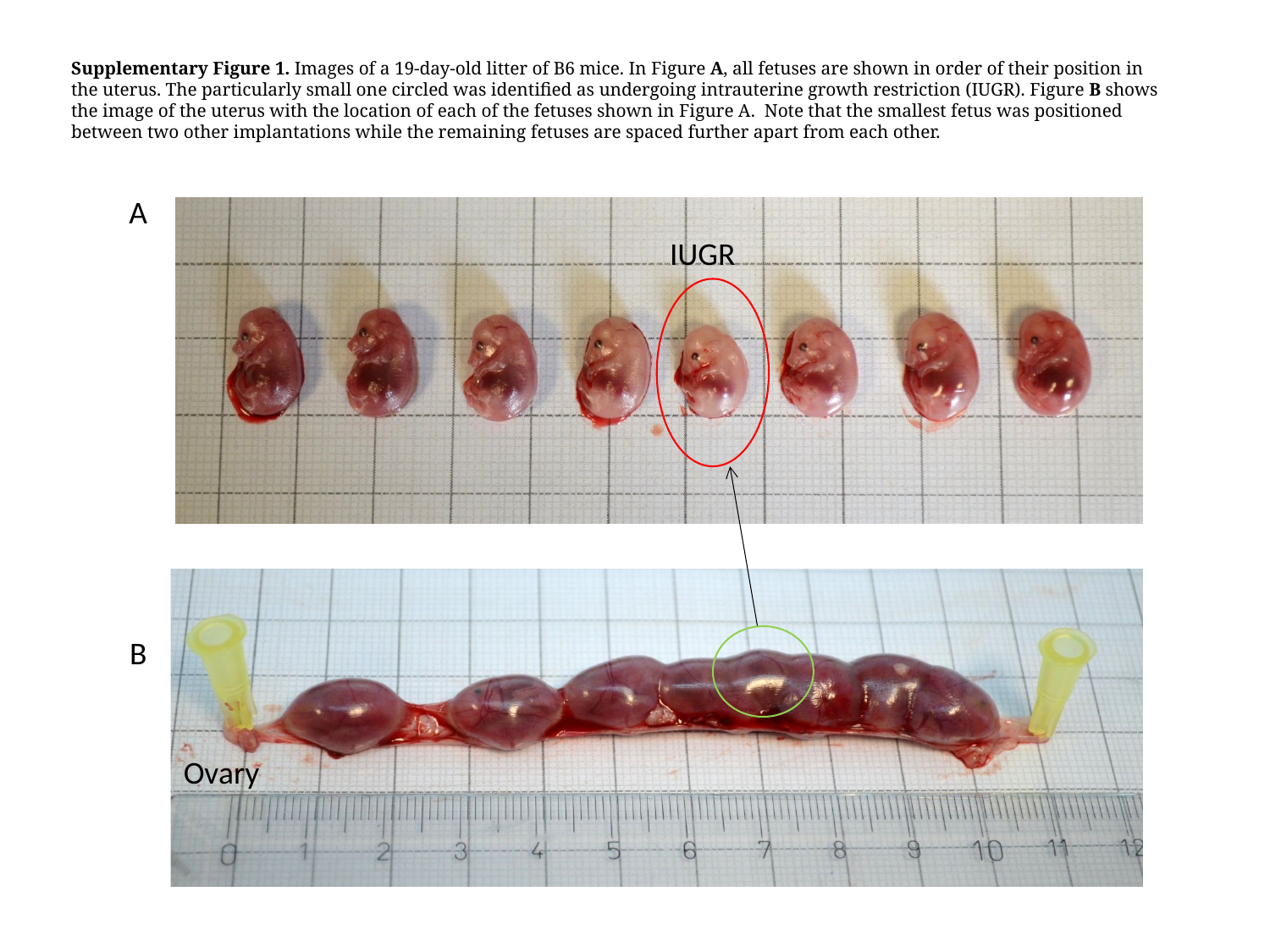

Supplementary Figure 1. Images of a 19-day-old litter of B6 mice. In Figure A, all fetuses are shown in order of their position in the uterus. The particularly small one circled was identified as undergoing intrauterine growth restriction (IUGR). Figure B shows the image of the uterus with the location of each of the fetuses shown in Figure A. Note that the smallest fetus was positioned between two other implantations while the remaining fetuses are spaced further apart from each other.
A
IUGR
B
Ovary
