# Supplementary material for: Postnatal Catch-Up Growth Programs Telomere Dynamics and Glucose Intolerance in Low Birth Weight Mice
Source: Int J Mol Sci. 2021 Apr 1;22(7):3657. doi: 10.3390/ijms22073657 (PMC8037520; doi:10.3390/ijms22073657)
Supplement: Supplementary file 1 [file ijms-22-03657-s001.zip › Supp Fig 2.pptx]

## Slide 1
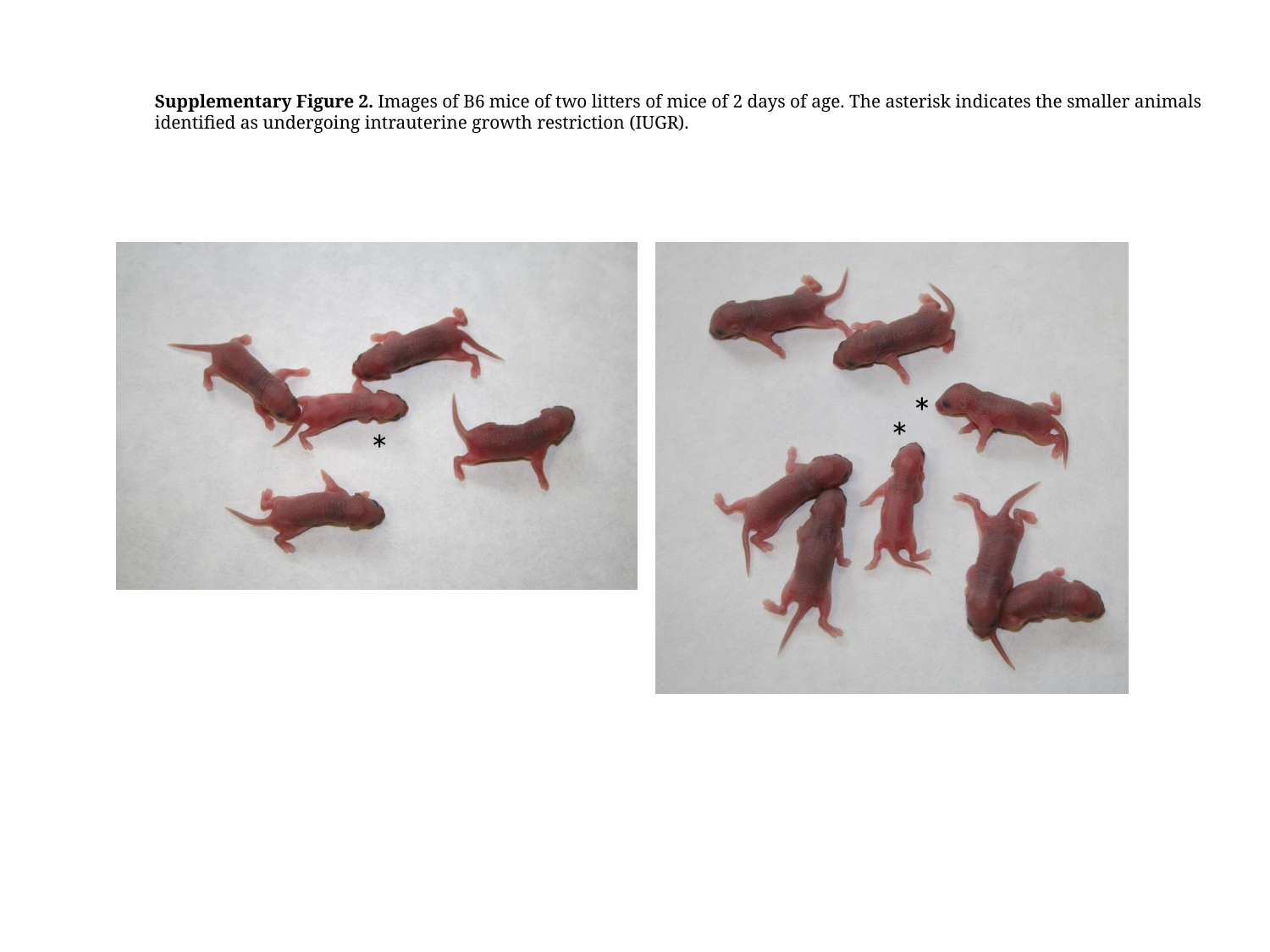

Supplementary Figure 2. Images of B6 mice of two litters of mice of 2 days of age. The asterisk indicates the smaller animals identified as undergoing intrauterine growth restriction (IUGR).
*
*
*
